# Interaction between acyl-ghrelin and BMI predicts clinical outcomes in hemodialysis patients

**DOI:** 10.1186/s12882-017-0442-8

**Published:** 2017-01-18

**Authors:** Ilia Beberashvili, Inna Sinuani, Ada Azar, Gregory Shapiro, Leonid Feldman, Keren Doenyas-Barak, Kobi Stav, Shai Efrati

**Affiliations:** 1 Nephrology Division, Assaf Harofeh Medical Center, Zerifin. Affiliated with the Sackler Faculty of Medicine, Tel Aviv University, Tel Aviv, Israel; 2 Department of pathology, Assaf Harofeh Medical Center, Zerifin. Affiliated with the Sackler Faculty of Medicine, Tel Aviv University, Tel Aviv, Israel; 3 Nutrition Department, Assaf Harofeh Medical Center, Zerifin. Affiliated with the Sackler Faculty of Medicine, Tel Aviv University, Tel Aviv, Israel; 4 Urology Department, Assaf Harofeh Medical Center, Zerifin. Affiliated with the Sackler Faculty of Medicine, Tel Aviv University, Tel Aviv, Israel

**Keywords:** Ghrelin, Acyl-ghrelin, BMI, Fat mass, Appetite, Hemodialysis, Survival, Inflammation

## Abstract

**Background:**

Ghrelin, a gastric orexigenic peptide, and body mass index (BMI) are known as inversely associated to each other and are both linked to cardiovascular (CV) risk and mortality in maintenance hemodialysis (MHD) patients. However, it is unclear whether the interaction between ghrelin and BMI is associated with a risk of all-cause and CV death in this population.

**Methods:**

A prospective observational study was performed on 261 MHD outpatients (39% women, mean age 68.6 ± 13.6 years) recruited from October 2010 through April 2012, and were followed until November 2014 (median follow-up-28 months, interquartile range-19–34 months). We measured acyl-ghrelin (AG) levels, appetite, nutritional and inflammatory markers, prospective all-cause and cardiovascular (CV) mortality.

**Results:**

During follow-up, 109 patients died, 51 due to CV causes. A significant interaction effect of high BMI and high AG (defined as levels higher than median) on all-cause mortality was found. Crude Cox HR for the product termed BMI x AG was 0.52, with a 95% confidence interval (CI): 0.29 to 0.95 (*P* = 0.03). Evaluating the interaction on an additive scale revealed that the combined predictive value of BMI and AG is larger than the sum of their individual predictive values (synergy index was 1.1). Across the four BMI-AG categories, the group with high BMI and high AG exhibited better all-cause and cardiovascular mortality irrespective of appetite and nutritional status (multivariable adjusted hazard ratios were 0.31, 95% CI 0.16 to 0.62, *P* = 0.001, and 0.35, 95% CI 0.13 to 0.91, *P* = 0.03, respectively). Data analyses made by dividing patients according to fat mass-AG, but not to lean body mass-AG categories, provided similar results.

**Conclusions:**

Higher AG levels enhance the favourable association between high BMI and survival in MHD patients irrespective of appetite, nutritional status and inflammation.

## Background

An inverse association between body mass index (BMI) and mortality, named the obesity paradox, has been established in different populations including maintenance hemodialysis (MHD) patients [1, 2]. Suggested reasons for this effect have been published and discussed elsewhere [2]. One reason given is better nutrition and as a result better short-term survival of MHD patients with higher BMI [2]. Among the factors contributing to the regulation of food intake, energy homeostasis and consequently to the regulation of body composition, ghrelin, predominantly a stomach-derived, 28-amino acid, orexigenic peptide, has a significant role [3, 4]. There are two major molecular forms of plasma ghrelin: acylated ghrelin (AG), with an n-octanoylated serine residue in position 3, and des-acyl ghrelin (DAG) [5]. AG accounts for about 10% of the total circulating ghrelin. In addition to its orexigenic properties, ghrelin has a multiplicity of physiological functions, affecting energy and glucose homeostasis, gastrointestinal, cardiovascular, pulmonary and immune functions, cell proliferation and differentiation, and bone physiology [6].

Although two to threefold higher plasma ghrelin levels in end-stage kidney disease (ESKD) patients have been reported in some studies, mainly on account of DAG [7, 8], conflicting results have been presented by others [9]. In this population, low plasma AG has demonstrated a significant association with cardiovascular morbidity [10] and low total ghrelin has been associated with all-cause and cardiovascular mortality risks, especially when considered in conjunction with nutritional status, inflammation and other weight-regulating hormones, such as leptin [11]. Ghrelin concentrations in MHD patients, like as in general population [12, 13], are inversely associated with body mass index and truncal fat mass [8]. Therefore, obese MHD patients with apparently a better prognosis [1, 2] will more likely have low concentrations of ghrelin which supposedly represent a poor prognosis in the same population [10, 11]. It is not at all clear if MHD patients with high BMI and high ghrelin levels will benefit more in terms of clinical outcome than MHD patients with high BMI and low ghrelin levels.

Therefore, the aim of this study is to investigate the interaction of AG with BMI for predicting all-cause and cardiovascular mortality in a cohort of MHD patients.

## Methods

### Patients

We have performed the prospective observational study. The study was approved by the local Ethics Committee (Assaf Harofeh Medical Center, Zerifin, affiliated with the Sackler Faculty of Medicine, Tel Aviv University, Israel). The study included MHD patients on hemodialysis treatment for at least 8 weeks, who were 18 years or older, and signed a local institutional review board-approved consent form. Patients with an anticipated life expectancy less than 6 months (e.g., because of a metastatic malignancy) were excluded. In total, 261 patients undergoing MHD treatment at our outpatient HD clinic and at two satellite HD clinics (from the same region), were included in the study. All patients underwent regular dialysis via their vascular access (58.2% of patients had arterio-venous fistula and 15.7%-arterio-venous grafts) 4 h three times per week at a blood flow rate of 250–300 ml/min and at a dialysis solution flow rate of 500 ml/min. The study population has been described in more detail in a recent publication [14]. In this same patient cohort, we observed an association between serum uric acid with various nutritional markers, muscle function, inflammation, health-related QoL and clinical outcomes. The patients were recruited from October 2010 through April 2012, and were followed until November 2014 or were censored (kidney transplantation or loss to follow-up). The median duration of the study was 28 months (interquartile range 19.0–34.0 months).

### Dietary intake and appetite assessment

The patients completed 3-day dietary histories (including a dialysis day, a weekend day and a non-dialysis day) as a food diary. Relying on these diaries the dietary energy and protein intake were calculated and normalized for ideal body weight according to the European best practice guidelines [15]. Ideal weight in the present study was calculated from the Lorentz equations differently for men and women. Dietary intake was calculated using computerized analysis (DOS-based program “MANA,” specially adapted for data entry and analysis of food intake records) [16].

Dietary protein intake was also approximated by determining normalized protein nitrogen appearance (nPNA) from the patient’s urea generation rate by using urea kinetics modeling [17]. Single-pool model urea kinetics was used to estimate the nPNA.

With respect to the self-reported appetite assessment, all patients were asked to grade their appetite during the past week according to a 5-point Likert scale: 1) very good, 2) good, 3) fair, 4) poor, and 5) very poor. These questionnaires were completed when blood samples were collected. The score was rearranged into two main groupings for further comparisons: diminished (combining fair, poor and very poor appetites) and non-diminished (combining very good and good appetites).

### Anthropometric measurements and handgrip strength

The following anthropometric variables were measured: BMI, triceps skinfold thickness (TSF), mid arm circumference (MAC), and calculated mid arm muscle circumference (MAMC).

The patients performed handgrip strength (HGS) in both the dominant and non-dominant arms using the Harpenden Handgrip Dynamometer (Yamar, Jackson, MI, USA). HGS was repeated three times and the highest value was noted.

### Nutritional scores

Overall nutritional assessment was performed using the malnutrition-inflammation score (MIS) [18] and the geriatric nutritional risk index (GNRI). GNRI was calculated using the equation developed by Bouillanne et al. [19] and modifying it by the nutritional risk index for elderly patients.

### Body composition analysis

Body composition was established by using body impedance analysis (B.I.A. Nutriguard-M, Data-Input, Frankfurt, Germany). We performed BIA within a half an hour post-dialysis according to the clinical application recommendations for analysis of bioelectrical impedance [20]. The multi-frequency technique (using 3 frequencies: 5, 50 and 100 kHz) were used to estimate the total body water (TBW), extracellular water (ECW), fat mass (FM) and lean body mass (LBM). These estimates were obtained using the NutriPlus software, version 5.1 (Data Input GmbH, Germany).

### Comorbidity index and clinical outcomes

We calculated the comorbidity index, developed recently by Liu et al. [21] and validated specifically for dialysis patient populations, as a measure of comorbid conditions.

Cardiovascular mortality was defined as death resulting from coronary heart disease, sudden death, stroke, or complicated peripheral vascular disease. Survival was determined from the day of examination.

### Laboratory evaluation

Predialysis blood samples and postdialysis serum urea nitrogen were obtained from non-fasting patients on a mid-week day. All biochemical analyses were measured by an automatic analyzer. Additionally, serum high sensitivity C-reactive protein (CRP) was measured by a turbidimetric immunoassay. AG, IL-6 and TNF-α levels were measured in plasma samples using commercially available enzyme-linked immunosorbent assay (ELISA) kits (R&D System, Minneapolis, MN, USA) according to the manufacturer’s protocol.

### Statistical analysis

Data are expressed as mean ± standard deviation (SD), or as median with interquartile range (IQR) for variables that did not follow a normal distribution, or as frequencies for categorical data.

To measure the differences between the variables in groups cross-classified by BMI and AG, a two-factor MANOVA with Wilks-lambda was used. Since the dialysis vintage, co-morbidity index, handgrip strength, CRP, IL-6 and TNF-α levels were not normally distributed, these variables were log transformed (lg_10_) before they were inserted into this model.

Survival analyses were performed using the Kaplan-Meier survival curve and the Cox proportional hazard model. The univariate and multivariate Cox regression analyses are presented as (HR; CI).

The interaction analyses between BMI and AG for predicting all-cause and cardiovascular mortality were investigated by Cox regression by simultaneously including into the multiple regression models BMI, AG and BMI x AG (BMI-based model). Other models to examine interactions between AG and fat mass, lean body mass, TSF and MAMC assembled in a similar manner. Interaction (synergism) between BMI and AG was defined as a deviation from additivity occurring when the observed hazard ratio (HR) for study outcomes of patients with both high BMI and high AG was higher than that expected by summing up the hazard ratio of those with high BMI and low AG or low BMI and high AG minus one [22].

All statistical analyses were performed using SPSS software, version 16.0 (SPSS Inc, Chicago, IL).

## Results

For 261 MHD patients at the start of the cohort, the median level of serum AG was 128.5 pg/ml, with IQR, 70.6 to 221.2 pg/ml. In our population, AG was inversely associated with BMI (r = −0.25, *p* < 0.001) and with waist circumference (r = −0.16, *p* = 0.03), as expected. To study the interactions between AG and BMI on clinical outcomes in our cohort, the patients were grouped according to AG and BMI levels. High and low concentrations were established according to median BMI and AG levels and cross-classified. The clinical and biochemical characteristics of the patients according to this categorization are detailed in Table 1. Patients with high AG levels had higher Kt/V and lower GNRI compared with the low AG group. Patients with high BMI had lower Kt/v and MIS, higher prevalence of DM, higher levels of albumin, creatinine, uric acid, body composition indicators (both, anthropometric and BIA derived) and GNRI, and fewer men and smokers were in this group than in the low BMI group. Significant BMI x AG interactions were found for gender, DM and serum cholesterol levels. Consequently, these variables were included in all further multivariable models. No statistically significant differences were evident between the groups in the use of medications (data not shown).

**Table 1:** Clinical and biochemical characteristics of 261 prevalent hemodialysis patients, grouped according to BMI and ghrelin levels^a^

	Low BMI (*n* = 131)		High BMI (n = 130)		
	Low AG (*n* = 51)	High AG (*n* = 80)	Low AG (*n* = 78)	High AG (*n* = 52)	MANOVA^b^
Clinical characteristics					
Age *(years)*	70.4 ± 14.8	67.9 ± 16.5	67.5 ± 10.9	69.8 ± 11.5	NS
Gender (men/women)^c^	80.4/19.6	60.3/39.7	57.5/42.5	50.0/50.0	B, B x AG
Log vintage *(months)*	1.26 ± 0.41	1.36 ± 0.40	1.19 ± 0.31	1.32 ± 0.35	NS
DM (yes)^c^	54.9	43.6	80.0	53.8	B, B x AG
Log comorbidity index	0.56 ± 0.33	0.55 ± 0.34	0.54 ± 0.34	0.47 ± 0.34	NS
Systolic BP (mm Hg)	142.0 ± 21.2	135.4 ± 27.8	140.6 ± 26.8	167.5 ± 87.2	NS
Diastolic BP (mm Hg)	65.0 ± 13.8	65.9 ± 13.8	66.5 ± 13.6	66.6 ± 12.9	NS
Kt/V	1.30 ± 0.25	1.44 ± 0.30	1.24 ± 0.31	1.32 ± 0.32	B, AG
Smoking (yes)^c^	19.6	20.5	6.3	9.6	B
Log handgrip strength (kg)	1.23 ± 0.31	1.21 ± 0.41	1.24 ± 0.35	1.20 ± 0.30	NS
Appetite (diminished)^c^	43.1	49.4	37.5	48.1	NS
Dietary intake					
DEI *(kcal/kg/d)*	23.9 ± 6.7	22.7 ± 6.0	24.2 ± 6.9	23.1 ± 4.9	NS
DPI *(g/kg/d)*	1.05 ± 0.31	1.02 ± 0.34	1.12 ± 0.32	1.11 ± 0.27	NS
nPNA *(g/kg/d)*	0.99 ± 0.27	1.03 ± 0.26	1.02 ± 0.22	1.07 ± 0.24	NS
Blood analysis					
Albumin *(g/L)*	37.9 ± 3.2	36.7 ± 4.4	38.1 ± 2.9	38.6 ± 3.5	B
Transferrin *(mg/dl)*	164.2 ± 26.4	169.5 ± 41.9	171.6 ± 25.7	168.7 ± 26.0	NS
Creatinine *(mg/dl)*	7.07 ± 2.0	6. 93 ± 2.02	7.39 ± 2.40	8.20 ± 2.02	B
Cholesterol *(mg/dl)*	148.0 ± 37.5	142.5 ± 31.0	143.5 ± 36.0	157.4 ± 39.1	B x AG
Triglycerides (mg/dl)	135.5 (93.0–167.0)	103.0 (82.0–140.3)	151.0 (107.5–238.0)	151.5 (113.5-194.8)	B, AG
Uric acid (mg/dl)	5.27 ± 0.86	5.52 ± 1.02	6.00 ± 1.20	6.23 ± 1.31	B
Hemoglobin *(g/dl)*	11.3 ± 1.2	10.9 ± 1.4	11.1 ± 1.1	10.8 ± 1.1	NS
Log TNF-α *(pg/ml)*	1.35 ± 0.42	1.35 ± 0.41	1.38 ± 0.39	1.35 ± 0.32	NS
Log CRP (mg/L)	0.76 ± 0.39	0.71 ± 0.47	0.80 ± 0.53	0.72 ± 0.59	NS
Log IL-6 *(pg/ml)*	0.97 ± 0.45	0.95 ± 0.53	1.01 ± 0.31	0.92 ± 0.37	NS
Anthropometric measurements					
TSF *(mm)*	12.6 ± 4.4	12.3 ± 4.3	18.7 ± 6.6	18.5 ± 6.1	B
MAC *(cm)*	25.5 ± 2.9	25.4 ± 3.0	30.6 ± 3.4	30.4 ± 3.6	B
MAMC *(cm)*	21.6 ± 2.5	21.7 ± 2.7	24.6 ± 2.9	24.4 ± 4.2	B
WC (cm)	94.1 ± 8.9	93.1 ± 11.7	115.7 ± 12.0	113.0 ± 10.5	B
W/H	0.99 ± 0.07	0.97 ± 0.10	1.05 ± 0.08	1.04 ± 0.08	B
Bioimpedance analysis					
ECW/TBW	0.37 ± 0.04	0.37 ± 0.07	0.42 ± 0.05	0.40 ± 0.05	B
FM *(kg)*	17.2 ± 5.3	16.7 ± 6.9	33. 8 ± 11.5	31.2 ± 8.3	B
LBM *(kg)*	45.7 ± 7.8	45.2 ± 9.1	54.1 ± 10.8	51.0 ± 9.8	B
Phase angle *(* ^*o*^ *)*	4.3 ± 1.0	4.6 ± 1.5	4.9 ± 1.4	4.8 ± 1.1	B
Nutritional scores					
MIS	7.28 ± 3.06	7.88 ± 3.99	5.21 ± 2.91	5.53 ± 2.95	B
GNRI	100.8 ± 7.4	98.6 ± 9.1	117.9 ± 9.0	115.2 ± 9.1	B, AG

*Abbreviations*: *AG* Acyl-ghrelin, *TNF-α* Tumor necrosis factor α, *DM* Diabetes mellitus, *BP* Blood pressure, *nPNA* Normalized protein nitrogen appearance, *IL-6* Interleukin-6, *BMI* Body mass index, *WC* Waist circumference, *W/H* Waist to hip ratio, *ECW/TBW* Extra-cellular water to total body water ratio, *FM* Fat mass, *LBM* Lean body mass, *MIS* Malnutrition-inflammation score, *GNRI* Geriatric nutritional risk index

^a^The low BMI or AG group was defined as BMI <26. 8 kg/m^2^ or Gh < 128.5 pg/ml - values below the medians of distribution

^b^Two-factor MANOVA. Significant (*P* < 0.05) effects are given for BMI (B), acyl-ghrelin (AG), and the interaction BMI with acyl-ghrelin (B x AG)

Continuous variables that did not follow a normal distribution (dialysis vintage, co-morbidity index, handgrip strength, TNF-α, CRP, IL-6) were log-transformed before their insertion into this model

^c^Assessed by χ^2^ test

We measured the prognostic value of AG combined with higher or lower BMI as well as with higher or lower levels of surrogate measures of body composition (Table 2). Both BMI and AG were significant predictors of all-cause mortality in adjusted models. A statistical interaction analysis showed a departure from a multiplicity of effects of high BMI (above the median) with high AG (above the median) levels. Crude Cox hazard ratio for all-cause mortality for the product termed BMI x AG was 0.52 with a 95% CI of 0.29 to 0.95 (*P* = 0.03). The hazard ratio for all-cause mortality remained significant after adjustments for age, sex, DM status, dialysis vintage co-morbidity index, smoking, Kt/V, cholesterol and IL-6. Interaction analyses carried out by stratifying patients according to fat mass-AG and TSF-AG, but not to lean body mass-AG, MAMC-AG or creatinine-AG categories, provided similar results (Table 2). No statistically significant interactions were observed between AG and body composition surrogate measures including BMI in predicting hazards for cardiovascular death.

**Table 2:** Crude and multiple Cox regression analysis of body composition surrogates, ghrelin and their interactions for predicting all cause and cardiovascular mortality in the study population (*n* = 261)

	Crude			Adjusted^a^		
Variable	HR	95% CI	p	HR	95% CI	p
*All-cause mortality*						
AG (>128.5 pg/ml)	0.73	0.50–1.07	0.10	0.67	0.45–1.00	0.05
BMI (>26.8 kg/m^2^)	0.62	0.42–-0.91	0.015	0.58	0.38–0.88	0.01
FM (>22.6 kg)	0.57	0.39–0.85	0.005	0.53	0.34–0.82	0.005
LBM (>48.7 kg)	0.83	0.56–1.23	0.35	0.88	0.54–1.42	0.59
Cre (>7.18 mg/dl)	0.50	0.34–0.73	<0.001	0.57	0.37–0.89	0.01
MAMC (>23.2 cm)	0.65	0.44–0.96	0.03	0.67	0.44–1.03	0.06
BMI*AG^b^	0.52	0.29–0.95	0.033	0.46	0.25–0.85	0.01
FM* AG	0.53	0.29–0.96	0.038	0.69	0.53–0.92	0.01
TSF* AG	0.42	0.23–0.78	0.006	0.61	0.45–0.82	0.001
LBM* AG	0.95	0.56–1.62	0.85	0.86	0.65–1.13	0.27
Cr* AG	0.87	0.50–1.52	0.63	0.81	0.62–1.05	0.11
MAMC* AG	0.96	0.96–1.02	0.45	0.79	0.58–1.06	0.12
*Cardiovascular mortality*						
AG (>128.5 pg/ml)	0.70	0.40–1.23	0.22	0.73	0.40–1.31	0.29
BMI (>26.8 kg/m^2^)	0.57	0.32–1.00	0.05	0.56	0.30–1.03	0.06
FM (>22.6 kg)	0.43	0.24–0.79	0.006	0.43	0.22–0.83	0.01
TSF (>14.0 mm)	0.62	0.35–1.07	0.09	0.76	0.40–1.44	0.39
LBM (>48.7 kg)	1.25	0.71–2.19	0.44	0.99	0.50–1.96	0.98
Cr (>7.18 mg/dl)	0.58	0.33–1.01	0.05	0.51	0.28–0.95	0.03
MAMC (>23.2 cm)	0.69	0.40–1.20	0.19	0.61	0.34–1.11	0.11
BMI* AG^b^	0.58	0.24–1.38	0.22	0.58	0.24–1.43	0.24
FM* AG	0.55	0.23–1.35	0.19	0.68	0.45–1.03	0.07
TSF* AG	0.59	0.26–1.38	0.23	0.73	0.50–1.08	0.12
LBM* AG	1.18	0.59–2.37	0.63	0.91	0.62–1.33	0.62
Cre* AG	0.67	0.30–1.47	0.31	0.68	0.46–1.01	0.06
MAMC* AG	0.69	0.31–1.55	0.37	0.66	0.43–1.01	0.06

*Abbreviations*: *CI* Confidence interval, *HR* Hazard ratio, *AG* Acyl-ghrelin, *BMI* Body mass index, *FM* Fat mass, *TSF* Triceps skinfold, *LBM* Lean body mass, *Cr* Creatinine, *MAMC* Midarm circumference calculated, *DM* Diabetes mellitus

^a^Adjusted for age, gender, DM status, dialysis vintage, comorbidity index, smoking, Kt/V and cholesterol

^b^Controlled for the main effects of variables included in interaction analysis

**Fig. 1:**
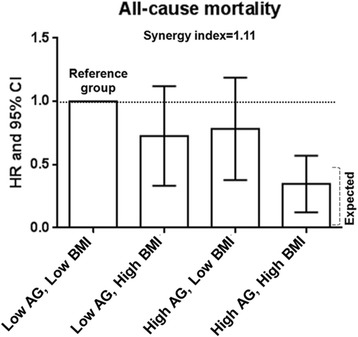
Interaction between BMI and acyl-ghrelin (AG) (below or above the corresponding median values) for explaining all-cause mortality. The data are expressed as hazard ratios (HR) and 95% confidence intervals (CI). The data are adjusted for age, sex, smoking, diabetes, co-morbidity index, dialysis vintage, Kt/V, and cholesterol. Deviation from additivity or the presence of interaction (synergism) was assessed by comparing the observed joint effect of high ghrelin and high BMI with that expected in the absence of interaction. The expected effect of high ghrelin and high BMI in the absence of interaction was calculated as HR_High AG, Low BMI_ + HR_Low AG, High BMI_ - 1

In addition to the multiplicative scale, we assessed the aforementioned interaction on an additive scale to verify that the combined predictive value of BMI and AG is larger than the sum of their individual predictive values. The excess risk for all-cause death caused by the interaction (synergy index) was 1.1 times higher than that portended by high BMI and high AG in the absence of an interaction (Fig. **1**).

**Fig. 2:**
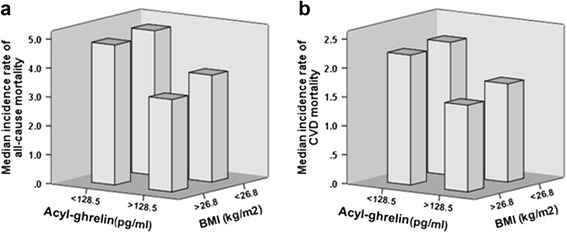
Interaction between BMI and acyl-ghrelin (AG) for explaining all-cause mortality (**a**) and CVD mortality (**b**). The patients are divided into four groups according to the median values of AG and BMI. The data are crude (unadjusted) incidence rates of all-cause and CVD mortality. Abbreviations: BMI, body mass index; CVD, cardiovascular disease

During the follow-up period (median-28 months), 109 patients died (19 deaths/100 person-years). Of the 52 patients in the high BMI-high AG group, 15 patients died (28.8%) in contrast to 28 out of 51 patients (54.9%) that died in the low BMI-low AG group (Table 3). Moreover, only seven cardiovascular deaths (13.5%) occurred in the high BMI-high AG group versus 15 cardiovascular deaths (29.4%) out of 51 patients that comprised the low BMI-low AG group. The association between BMI and the incidence rate of all-cause and CV mortality was closely dependent on AG categories (effect modification of BMI by ghrelin), with the incidence rate of all-cause and CV mortality being maximal in patients with lower BMI and lower AG and minimal in patients with higher BMI and higher AG (Fig. **2**). Further data adjustment did not substantially affect these results (Table 4). The hazard for death of patients with higher BMI and AG was 0.31 (95% CI: 0.16–0.62) for all-cause death and 0.35 (95% CI: 0.13–0.91) for cardiovascular death, after multivariate adjustments.

**Table 3:** Individual causes of death grouped according to BMI and AG levels^a^

	Low BMI (*n* = 131)		High BMI (*n* = 130)	
	Low AG (*n* = 51)	High AG (*n* = 80)	Low AG (*n* = 78)	High AG (*n* = 52)
Myocardial infarction	5	1	2	1
Cerebrovascular accident	3	2	3	1
Peripheral vascular disease	4	3	2	1
Sudden death	1	6	4	1
Other cardiovascular	2	4	2	3
Infection/septicemia	6	13	6	7
Surgical peritonitis	1	1	3	-
Cancer	4	1	2	-
Other	2	6	5	1
All deaths^b^, n (%)	28 (54.9%)	37 (47.4%)	29 (36. 2%)	15 (28.8%)
CVD deaths^b^, n (%)	15 (29.4%)	16 (20.5%)	13 (16.2%)	7 (13.5%)

*Abbreviations*: *CVD* Cardiovascular disease, *BMI* Body mass index, *AG* Acyl-ghrelin

^a^Indicated as the causes and number of deaths (n) in each category

^b^Indicated as the number of deaths and percentage, expressed as a proportion of the total number of patients in the group. The proportion of deaths was higher in the low BMI combined with low acyl-ghrelin group as assessed by χ^2^ test (*P* < 0.001 for all deaths and CVD deaths)

**Table 4:** Crude and adjusted^a^ all-cause and CVD-related mortality grouped according to BMI and ghrelin levels^b^

Variable		All-cause mortality		Cardiovascular mortality	
		HR (95% CI)	P	HR (95% CI)	P
Low BMI, low AG (*n* = 51)	*Crude*	1.0		1.0	
	*Adjusted*	1.0^c^		1.0^c^	
Low BMI, high AG (*n* = 80)	*Crude*	0.72 (0.44–1.19)	0.20	0.58 (0.29–1.19)	0.14
	*Adjusted*	0.71 (0.42–1.23)	0.23	0.64 (0.29–1.39)	0.26
High BMI, low AG (*n* = 78)	*Crude*	0.64 (0.38–1.08)	0.10	0.52 (0.29–1.10)	0.09
	*Adjusted*	0.63 (0.35–1.11)	0.11	0.51 (0.22–1.15)	0.10
High BMI, high AG (*n* = 52)	*Crude*	0.36 (0.19–0.68)	0.002	0.33 (0.13–0.82)	0.02
	*Adjusted*	0.31 (0.16–0.62)	0.001	0.35 (0.13–0.91)	0.03

*Abbreviations*: *CI* Confidence interval, *HR* Hazard ratio, *CVD* Cardiovascular disease in the past, *BMI* Body mass index, *AG* Acyl-ghrelin

^a^Adjusted for age, gender, DM status, dialysis vintage, co-morbidity index, smoking, Kt/V, cholesterol and IL-6

^b^The low BMI or AG group was defined as BMI <26. 8 kg/m2 or Gh < 128.5 pg/ml - values below the medians of distribution

^c^The group of patients who had low BMI (defined as BMI levels below median) and low acyl-ghrelin (defined as acyl-ghrelin levels below median) was used as a reference

## Discussion

Our study provides evidence that MHD patients with higher BMI combined with higher AG concentrations have a diminished all-cause and cardiovascular-related mortality risk. Since both ghrelin [10, 11] and BMI [1, 2] are associated with survival in the same directions, it is possible that the combination of high BMI and high AG levels confers a decreased risk of mortality in MHD patients by their cumulative independent contributions to mortality. To our knowledge, the association of interaction of BMI and AG levels with survival of HD patients has not been previously investigated.

As a biomarker for mortality in the ESKD population, ghrelin has been described as associated with traditional cardiovascular risk factors, inflammation, and PEW [23]. The existing experimental data based on rodent heart failure models provides evidence to suggest that ghrelin may lower the risk of mortality and improve cardiovascular outcomes [24]. AG attenuates *in vitro* angiogenesis induced by oxidized low-density lipoprotein in human coronary artery endothelial cells [25]. Ghrelin also suppresses pressure overload-induced cardiac hypertrophy [26], reverses endothelial dysfunction in patients with metabolic syndrome by increasing nitric oxide productions and bioactivity [27], suppresses sympathetic activity and attenuates left ventricular remodeling following myocardial infarction in Sprague-Dawley rats [28]. In clinical studies, lower ghrelin levels have been shown to be independent predictors of acute ischemic stroke [29], as well as markers of acute and early myocardial infarction recovery periods [30]. In the MHD population, low plasma ghrelin has been linked to cardiovascular morbidity [10] and all-cause and cardiovascular mortality risks [11]. Lower all-cause and cardiovascular mortality rates are attributable to overweight and obese MHD patients compared to MHD patients with lower BMI [2]. While these relationships can be modified by several factors such as inflammation [31] or serum creatinine [32], the linear inverse relationship between BMI and mortality was found as robust across models including marginal structural model analyses [33]. It is possible that a wide array of cardiovascular activities in both physiological and pathophysiological states enable AG to enhance the positive association between high BMI and better clinical outcomes in the MHD population. The finding that the lowest percentage of cardiovascular deaths during follow-up was in the high BMI-high AG category, allows this assumption.

Interestingly, AG excess can contribute to obesity-associated insulin resistance in metabolic syndrome in general population [34]. The prevalence of metabolic syndrome is high in ESKD patients [35]. Therefore, in the investigation of underlying pathophysiology of BMI-AG interaction, the association between AG and metabolic syndrome should be considered. While not all markers of the metabolic syndrome were measured in our study, no statistically significant differences were observed in BMI, waist circumference, blood pressure, triglycerides and uric acid levels between the high BMI-high AG group compared with the three other groups categorized by BMI and AG. Furthermore, in view of the high prevalence of DM in our population, we adjusted for diabetes in all multivariable models used in our study. These data allow us to rule out the association of AG with metabolic syndrome as the basis of interaction between AG and BMI observed in our population.

Ghrelin has potent anti-inflammatory effects [36–38] that might play a role in the described interaction between BMI and AG on the clinical outcome of MHD patients. The impact of chronic inflammation on the low survival rate in the MHD population has been previously documented [39, 40]. Ghrelin inhibits proinflammatory cytokine release from T cells and monocytes [36], and has been shown to suppress nuclear factor-κB activation in human endothelial cells in vitro and endotoxin-induced cytokine production in vivo [37]. In addition, ghrelin treatment in a rat model of CKD has resulted in a decrease of circulating inflammatory cytokines [38]. We however did not observe any statistically significant differences in inflammatory markers measured in our study across the four BMI-AG groups. Furthermore, combined use of ghrelin and inflammatory markers (IL-6, CRP) failed to explain the association between the metabolic syndrome and the cardiovascular mortality in older adults [41]. Taken together, the anti-inflammatory properties of ghrelin might be a factor but are probably not the explanation for the interaction between BMI and AG on survival rate in our study.

As an appetite-related hormone, high concentrations of AG may contribute to a better nutritional status in MHD patients, even in those with higher BMI. This could be a potential explanation for our results. Certainly, while elevated BMI characterizes a better nutritional condition when compared to normal BMI [42] and accordingly, improved survival rates [1, 2] in MHD patients, obese sarcopenia was found to be associated with a poor prognosis in this population [43]. It is possible that overweight and obese ESKD patients with higher ghrelin are less likely to present with PEW. DeBoer et al. [38] have shown that ghrelin administration over a 2-week period increased lean body mass retention in rats with cachexia associated with CKD. Low total ghrelin levels have been related to worsening nutritional status in a two-year follow-up period in the elderly [44]. The statistically significant increase in fat-free mass accompanied by improvements in muscle strength has been shown in a two-year clinical trial of an oral ghrelin mimetic in healthy individuals [45]. Short-term AG administration results in a significant increase in dietary intake in malnourished MHD [46] and peritoneal dialysis [47] patients. Although BMI displayed the previously described positive associations with nutritional markers [42] in our cohort, no statistically significant differences in nutritional characteristics, including lean body mass, were observed in the high BMI and high AG group compared with the three other groups, categorized by BMI and AG. The only nutritional marker that exhibited statistical significance in terms of BMI and AG interactions was the serum cholesterol concentration that was higher in the high BMI- high AG group. However, this interaction wasn’t independent and the inclusion of the cholesterol levels in multivariable models didn’t influence the results of our study. In addition, interaction analyses carried out on multiplicative scale revealed no interaction for lean body mass, MAMC or serum creatinine with AG on clinical prognosis of our population. Furthermore, no statistically significant differences in appetite and consequently dietary intake between the four BMI-AG groups were observed in our study. The complexity of appetite regulation in MHD patients which involves counter-regulatory signaling of orexigens (ghrelin, neuropeptide Y) and anorexigens (leptin, peptide YY) [48] may explain this finding. The lack of a statistical difference in the nutritional parameters of the high BMI and high AG group compared with the other BMI-AG categories may suggest that the biological basis for statistical interaction between the fat mass and AG on the survival in our study is the fat tissue’s property to secrete adipokines [49]. There are dual competing effects (protective - due to nutrition and deleterious - due to inflammatory adipokines) of fat mass on survival in MHD patients [50]. We believe that the favorable balance between the various pro- and anti-inflammatory adipokines may enhance AGs’ effect on clinical outcomes in MHD population with higher BMI, presumably through modulating insulin resistance and the cardiovascular effects of AG. This hypothesis should be tested in future studies.

Our study has limitations that should be considered. First, no definitive cause-and-effect relationship can be derived for any of the analyzed risk factors. This limitation is typical for observational approach study. Second, we might have underestimated the cardiovascular deaths proportion. This is because the cause of death was taken from the patient records and was not postmortemly confirmed. Third, samples were taken in non-fasting conditions, 1–3 h after a meal. It is difficult to obtain fasting blood samples from patients with diabetes and patients with an afternoon or night dialysis schedule. On the other hand, AG levels were characterized by a blunted premeal rise in dialysis patients [48] and less markedly affected by the postprandial state when compared with healthy controls [51]. Another limitation is assessment of dietary intake by a 3 day food record, as results can be subjective and incomplete. Our study did not have detailed serum markers of oxidative stress, insulin resistance or oxidized lipids, which could have provided additional evidence to reinforce proposed mechanistic pathways for our findings of strong associations. Finally, AG measurements in our study didn’t account for the parallel evaluation of some of the pro- and/or counter-regulatory molecules to AG’s actions such as adipokines that are also involved in appetite and body composition regulation [49, 50]. Despite these limitations, the availability of extensive data of nutritional and inflammatory biomarkers, body composition, comorbidities, and long-term follow-up strengthens the study.

## Conclusions

In conclusion, we report that higher AG levels enhance the favorable association between high BMI and survival in MHD patients irrespective of appetite, nutritional status and inflammation. Apparently, the role of AG as a cardiovascular marker contributes to this interaction. While the potential clinical utility of AG therapy in MHD patients in reversing PEW and associated worse prognosis has been previously discussed [11], based on the results of our study, overweight and obese MHD patients without PEW but with low AG levels, may also benefit from ghrelin therapy to improve long-term outcomes. The subcutaneous administration of 3.6 nmol/kg AG once a day (according to the short-term studies performed in healthy volunteers [52] and in MHD or PD patients [46, 47]), given 1 h before a meal, presumably for a period between 6 months to a year, may serve this purpose. Concomitantly, the risks of developing ghrelin resistance and its mitogenic potential should also be considered [9].

## Abbreviations

AG: Acyl-ghrelin
BMI: Body mass index
CI: Confidence interval
CVD: Cardiovascular disease in the past
DM: Diabetes mellitus
DM: Diabetes mellitus
ECW/TBW: Extra-cellular water to total body water ratio
ESKD: End stage kidney disease
FM: Fat mass
GNRI: Geriatric nutritional risk index
HR: Hazard ratio
IL-6: Interleukin-6
LBM: Lean body mass
MAC: Mid arm circumference
MAMC: Midarm circumference calculated
MHD: Maintenance hemodialysis
MIS: Malnutrition-inflammation score
nPNA: Normalized protein nitrogen appearance
PEW: Protein-energy wasting
TNF-α: Tumor necrosis factor α
TSF: Triceps skinfold
W/H: Waist to hip ratio
WC: Waist circumference
